# Identifying genes and regulatory pathways associated with the scleractinian coral calcification process

**DOI:** 10.7717/peerj.3590

**Published:** 2017-07-20

**Authors:** Eldad Gutner-Hoch, Hiba Waldman Ben-Asher, Ruth Yam, Aldo Shemesh, Oren Levy

**Affiliations:** 1 Department of Zoology, The George S. Wise Center for Life Sciences, Tel Aviv University, Tel Aviv, Israel; 2 The Interuniversity Institute for Marine Sciences, Eilat, Israel; 3 The Mina and Everard Goodman Faculty of Life Sciences, Bar-Ilan University, Ramat Gan, Israel; 4 Department of Earth and Planetary Sciences, Weizmann Institute of Science, Rehovot, Israel

**Keywords:** Scleractinian coral, Calcification, Regulatory pathways, Wnt pathway, TGF-beta/BMP pathway, Microarray, Gene expression

## Abstract

Reef building corals precipitate calcium carbonate as an exo-skeleton and provide substratum for prosperous marine life. Biomineralization of the coral’s skeleton is a developmental process that occurs concurrently with other proliferation processes that control the animal extension and growth. The development of the animal body is regulated by large gene regulatory networks, which control the expression of gene sets that progressively generate developmental patterns in the animal body. In this study we have explored the gene expression profile and signaling pathways followed by the calcification process of a basal metazoan, the Red Sea scleractinian (stony) coral, *Stylophora pistillata*. When treated by seawater with high calcium concentrations (addition of 100 gm/L, added as CaCl_2_.2H_2_O), the coral increases its calcification rates and associated genes were up-regulated as a result, which were then identified. Gene expression was compared between corals treated with elevated and normal calcium concentrations. Calcification rate measurements and gene expression analysis by microarray RNA transcriptional profiling at two time-points (midday and night-time) revealed several genes common within mammalian gene regulatory networks. This study indicates that core genes of the Wnt and TGF-β/BMP signaling pathways may also play roles in development, growth, and biomineralization in early-diverging organisms such as corals.

## Introduction

Coral calcification is a process of skeletal elements extension and is accompanied by the development of the coral’s tissues. In general, the biomineralization and development processes are generated by the differential gene expressions with specialized cellular properties, as demonstrated in various organisms (Belcher et al., 1996; Gardner et al., 2011; Kuballa & Elizur, 2008; Reyes-Bermudez et al., 2009). These gene expression characteristics of many cellular processes, among them are biomineralization and tissue growth, which are coordinated by networks of regulatory genes.

The process of skeleton growth in corals has been suggested to be controlled by a specialized tissue called calicoblastic epithelium, which is comprised of ectodermal cells within the aboral coral layer, opposite the oral side (Gattuso, Allemand & Frankignoulle, 1999). The transport of calcium ions to the site of the growing skeleton is one feature for characterizing the controlled process of coral calcification. The mechanism behind the transport of calcium is under debate, and three pathways have been proposed: (i) active transcellular transport of calcium through calicoblastic cells, (ii) passive paracellular diffusion of calcium or seawater between calicoblastic cells, and (iii) a combination of transcellular and paracellular pathways (Reviewed in Allemand et al., 2011 and Allison et al., 2014).

Another aspect of the controlled process is the molecular mechanism which is estimated to include genes in several molecular pathways which aid the skeletogenesis. Various genes have been identified to be associated with the calcification mechanism and are involved in the transport of CaCO_3_ reactants. The plasma membrane calcium-activated ATPase gene was isolated and cloned from a coral cDNA and ESTs (Zoccola et al., 2004; Zoccola et al., 2015), and its activity was measured and characterized (Furla, Allemand & Orsenigo, 2000; Ip, Lim & Lim, 1991; Isa, Ikehara & Yamazato, 1980). A voltage-dependent calcium channel was cloned and sequenced from cDNA, and its protein product was labeled within the calicoblastic ectoderm (Zoccola et al., 1999). Another gene that is involved in the transport of CaCO_3_ reactants is the carbonic anhydrase, which has been identified and characterized by molecular biology, biochemistry and pharmacology techniques (reviewed in Bertucci et al., 2013). Several genes involved in the calcification mechanism have also been discovered as proteins found in the coral’s organic matrix with protein extraction, separation and characterization techniques (Drake et al., 2013; Ramos-Silva et al., 2013), or with RNA extraction and transcriptome assembly methods (Mass et al., 2013; Moya et al., 2012). The coral’s organic matrix proteins are characterized as highly acidic proteins that are abundant with aspartic and glutamic acids. Still, the molecular aspects of the skeletogenesis process are not fully understood.

The calcification process is affected by several factors: temperature, light, nutrients, food availability (Langdon & Atkinson, 2005). Another important element affecting calcification rates of corals is the calcium carbonate saturation state of the mineral aragonite (Cohen et al., 2009; Gattuso et al., 1998; Marshall & Clode, 2002). When Ω_arag_ exceeds the value of 1 (Ω_arag_ ≥ 1), CaCO_3_ precipitation is thermodynamically favored. Following the concept that seawater Ω_arag_ is a function of CO${}_{3}^{2-}$ and calcium ion ([Ca^2+^]) concentrations (Cyronak, Schulz & Jokiel, 2016), Longdon et al. (2000) and Marshall & Clode (2002) showed that exposing scleractinian corals to seawater with high calcium concentrations induces high calcification rates.

Exploring the calcification process of scleractinian corals is valuable in several ecological aspects: it is responsible for a global source of atmospheric carbon dioxide (CO_2_) in the tropical seas (Frankignoulle, Canon & Gattuso, 1994; Borges, Bruno & Frankignoulle, 2005), establishment of habitats for prosperous marine life (Hoegh-Guldberg et al., 2007; Knowlton, 2001) and for paleoenvironmental records (Mitsuguchi et al., 1996; Roberts, Wheeler & Freiwald, 2006).

The aim of this study is to identify potential key genes and pathways that are related to the calcification apparatus. This will be achieved by exposing the coral to seawater with high calcium concentrations to induce an alteration in gene expression profiles, identified using a DNA microarray analysis. In addition, we explored the differential genes expressions between day and night in the presence of high calcium concentration seawater in order to identify the presence of networks of regulatory genes in a basal metazoan.

## Materials and Methods

### Coral collection

Scleractinian coral colonies of *Stylophora pistillata* were collected by SCUBA diving from 3–6 m depth at the coral reef near the Inter-University Institute (IUI) in Eilat, Aqaba Gulf, Red Sea. The branching coral colonies were fragmented to 5–10 cm long nubbins and transferred to an outdoor shaded running seawater table at the IUI for a month acclimation and recovery before the experiment started. The Israeli Nature and National Parks Protection Authority approved the collection of corals in this study (permit No. 2009-35556).

### Calcification rates measurements under different seawater calcium concentrations

High calcium seawater was prepared by adding either 100 mg/L or 200 mg/L calcium (added as CaCl_2_⋅2H_2_O) to filtered seawater (0.45 µm).

**Figure 1:**
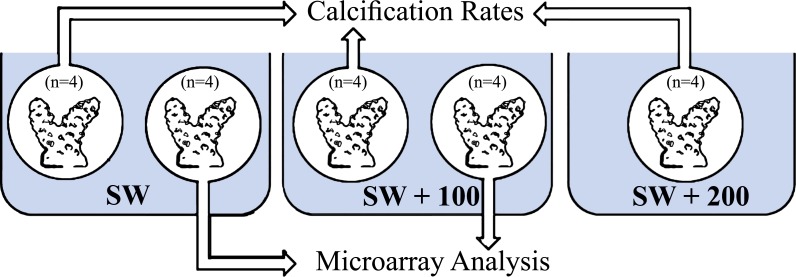
Experimental layout design. Eight fragments of *Stylophora pistillata* were incubated in calcium seawater (SW) concentrations of seawater plus 100 mg/L calcium (SW + 100) and plus 200 mg/L calcium (SW + 200) during daytime (11:00) and during night-time (23:00). In each sampling time, four replicates were analyzed for calcification rates and four replicates from SW and SW+100 treatments, for microarray analysis.

Four coral fragments of *S. pistillata*, for each treatment (Fig. 1), were incubated in running seawater tables for 1.5 h for two time points: midday (11:00) and night-time (23:00). The fragments were incubated separately in three seawater calcium concentrations: natural calcium concentration (SW), natural calcium concentration added with 100 mg/L (SW + 100) and natural calcium concentration added with 200 mg/L (SW + 200).

The calcification rate values (µmol CaCO_3_ h^−1^ cm^−2^) were calculated according to the equation:

\begin{eqnarray*} \frac{ \frac{\Delta {A}_{T}}{2} \ast ({V}_{\mathrm{chamber}}-{V}_{\mathrm{coral}})}{{T}^{\ast }{A}_{\mathrm{coral}}} \end{eqnarray*}

where Δ*A*_*T*_ is the difference in Total Alkalinity (*A*_*T*_) measured between the beginning and the end the incubation period, *V* is the volume of the chamber or the coral fragment, *T* is the duration of the incubation and *A* is the coral surface area. The coral’s surface area (*A*) was calculated by considering the branched fragments as perfect cylinders, hence the height and diameter were measured and used for the cylinder-coral fragment surface area calculation (Mass et al., 2007). Such short period incubation was already been successful for distinguishing differences of calcification rates between treatments (Cohen, Dubinsky & Erez, 2016; Gutner-Hoch et al., 2016).

### Microarray experiment set-up and coral fragments sampling

Four coral fragments of *S. pistillata* were incubated for 1.5 h in each of the two different calcium concentrations (SW, SW + 100), and for two time points: in the mid-day (11:00) and at night-time (23:00). After incubation, coral fragments were sampled, snap froze in liquid nitrogen and stored in −80 C until further handled.

**Figure 2:**
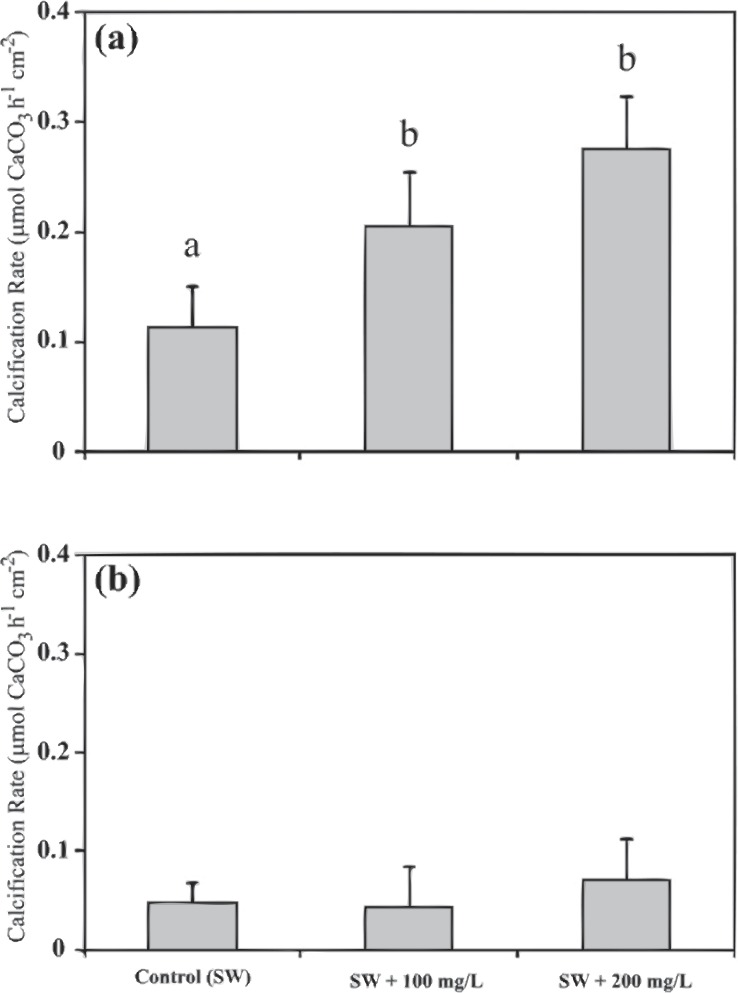
Calcification rates of *Stylophora pistillata* under different calcium seawater (SW) concentrations of seawater plus 100 mg/L calcium and plus 200 mg/L calcium during 11:00 (A) and during 23:00 (B). Calcium supplemental was added as CaCl_2_⋅2H_2_O. Means followed by the same letter are not significantly different, one-way ANOVA followed by Tukey’s post-hoc test. Error bars represent SD. *n* = 4.

The coral fragments that have been subjected to SW + 200, were removed from gene expression analysis because no statistically significant was found between the calcification rates of SW + 100 and SW + 200 treatments (Fig. 2).

### RNA extraction and microarray design

Total RNA was isolated from the *S. pistillata* fragments by using TRIzol^®^ reagent (Invitrogen, Carlsbad, CA, USA) according to manufacturer’s instructions. RNA quantity and integrity were assessed with a NanoDrop ND-1000 spectrophotometer and an Agilent 2100 Bioanalyzer, respectively. Total RNA was labeled and hybridized against custom *S. pistillata* microarray; an Agilent two-color gene expression microarray platform with 8 × 15 K probe per slide; microarray samples included four SW+100 and four SW (control) samples for each time point (11:00 and 23:00). Each treatment was combined with a differently labeled control sample from the same time point for hybridization to each compartment of the microarray slide. Oligonucleotide probes (60-mers) were designed based on approximately 12,000 genes predicted to encode proteins retrieved from a recent de novo assembly of 454-sequenced EST libraries of *S. pistillata* (Karako-Lampert et al., 2014). Labeling and hybridization were conducted using the Agilent Low Input Quick Amp Labeling Kit according to the manufacturer’s instructions. The intensity of the emitted fluorescence from a target spot on the array was detected using an Agilent G2565BA microarray scanner. The raw data as well as the processed data of the microarray were deposited under Gene Expression Omnibus (GEO) (http://www.ncbi.nlm.nih.gov/geo) accession number GSE87159. *Stylophora pistillata* EST data are also stored at the Cnidarian Database of Centre Scientifique de Monaco: http://data.centrescientifique.mc/CSMdata-home.html.

### Microarray results analysis

The data from all arrays were first subjected to background correction and LOESS within-array normalization using Agilent Feature Extraction software (version 9.5.1.1; Agilent Technologies, Santa Clara, CA, USA). The remaining analyses were performed in Partek^®^ Genomics Suite software (version 6.6; Partek Inc., St. Louis, MO, USA). Data from three biological replicates were used to perform a one-way ANOVA. The quintile normalized data were analyzed to identify genes with significantly up- or down-regulated expression (FDR *p*-value < 0.05) with an arbitrary cutoff of at least a two-fold change. As the microarray contains a diversity of genes originated from different organisms, all genes were transformed to *Homo sapiens* ones. Networks of highly interconnected proteins were generated using the STRING (Search Tool for the Retrieval of Interacting Genes, Heidelberg, Germany) 9.0 database (Szklarczyk et al., 2011) and GeneMania (Warde-Farley et al., 2010). The gene function clustering in GeneMania was predicted by using the Gaussian field label propagation algorithm. For this study the *Homo sapiens* dataset of functional association network was used in a network-weighting method of ‘Gene-Ontology (GO) based weighting, biological process based. The names of samples within the microarray analysis were labeled C11 and T11 as for control (reference) and treatment at daytime (11:00), respectively. The samples C23 and T23 were labeled as for control (reference) and treatment for night-time (23:00), respectively.

### Semi-quantitive real time PCR

The relative expression of genes was analyzed through Qiagen Corbett machine, using a two step real-time PCR. The gene expression from every sample was amplified in triplicates, with a single volume of 10 µl containing: 3 µl of diluted cNDA with 0.5 µl of primer set (see Table 1 for primer sequences), 1.5 µl double distilled water (DDW) and 5 µl of GoTaq qpcr Master Mix Kit (Promega, USA). Cycle threshold values for each time point were compared to the internal reference genes, according the 2^−^^ΔΔ^^CT^ method (Livak & Schmittgen, 2001). The gene *β*-*actin* was used for normalization in all RT-PCR analyses (Levy et al., 2007).

**Table 1:** Primer list for *S. pistillata*’s real-time PCR analysis.

Gene name	Directions	Sequence	Product length (bp)
*β-actin*	Forward	TACGTTGCCATCCAAGCTGTACTT	108
	Reverse	TCCTTCATAGATTGGTACAGTGTGGC	
*smad1*	Forward	TTTTCAACAGCCAAGAATTTGCCCAG	139
	Reverse	ATCTTGGCGGTGATACTCAGCTC	
*galaxin*	Forward	TGTGATGCCAACCCACATTCAAAGATT	146
	Reverse	ATGTCACTCCCACAACAGGCTGATT	
*galaxin-like*	Forward	GGACGGTTTGTAATTTATTGACAGCCAGG	139
	Reverse	GATCTGCCATGTTCAGGGAGTTTGTCT	

### Statistical analyses

For statistical analyses, one-way ANOVA was used and followed by Tukey HSD (honestly significant differences) to assess the differences in experimental treatments of calcification rates and genes’ real-time PCR results. All statistical analyses were conducted using SPSS 20.0 (IBM, USA), and the results were considered statistically significant at *p* < 0.05.

## Results

### Calcification rates of *S. pistillata* under increased seawater calcium concentration

Calcification rates of the Red Sea scleractinian coral *Stylophora pistillata* during daytime (11:00) were enhanced by 54% and 104% when the seawater calcium concentration was increased by adding 100 mg/L and 200 mg/L (of CaCl_2_⋅2H_2_O), respectively. During night-time (23:00), calcification rates were not influenced by the calcium concentration additions (Fig. 2 and File S1). Statistically significant differences were found during the daytime between calcification in natural seawater and calcification with additional calcium concentration (one-way ANOVA *F*(2, 9) = 5.277, *p* = 0.03; *n* = 4), while during night-time no difference was found (one-way ANOVA *F*(2, 9) = 1.052, *p* = 0.39; *n* = 4).

### Microarray expression analysis

**Figure 3:**
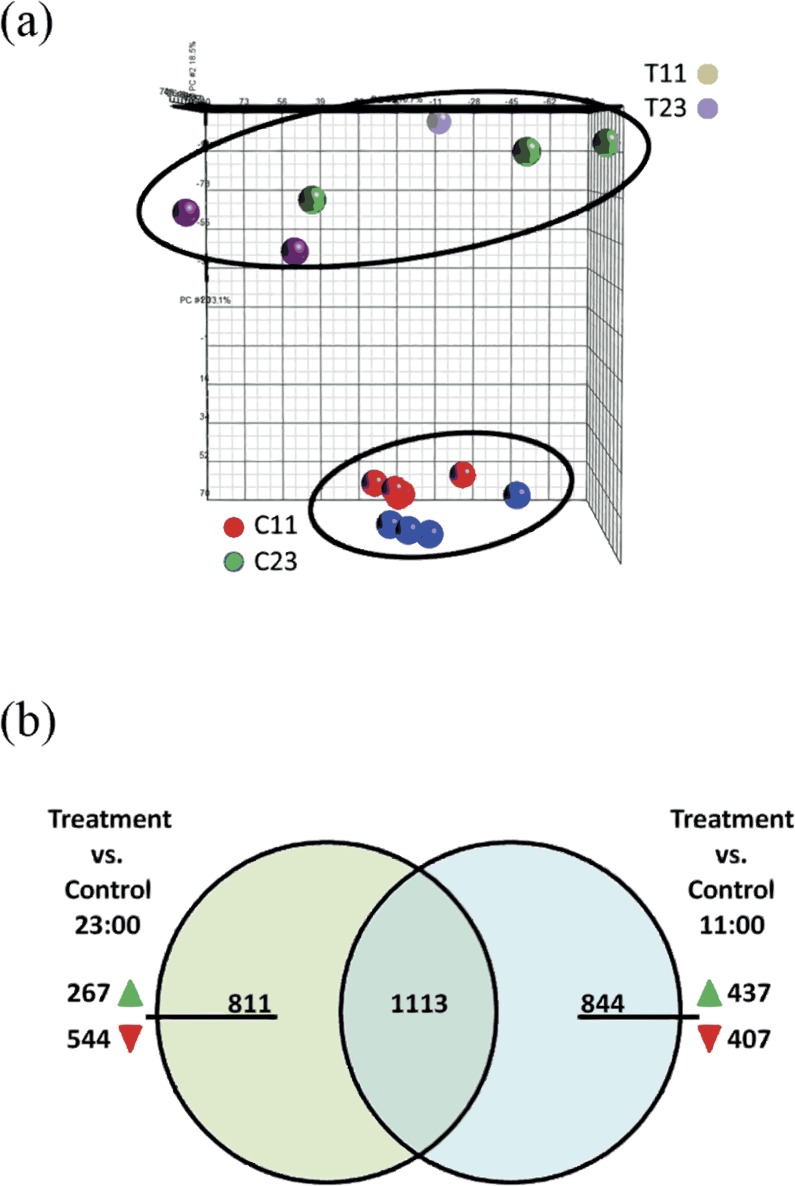
(A) Principle component analysis (PCA) map of samples from control 11:00, control 23:00, treated 11:00, and treated 23:00 (C11, C23, T11, and T23, respectively). The samples were applied to the PCA function of Partek. Analysis show distinct separation between treated and control samples. (B) Venn diagram representing the overlap of the total of 2,768 genes of the total 2,768 differentially expressed genes at treatment samples from 11:00 and from 23:00. Among the 844 unique genes that were significantly up- or down-regulated expression during the day, 437 were showed up-regulation of their expression and 407 were down-regulated. Among the 811 unique genes that were significantly up- or down-regulated expression during the night, 267 were showed up-regulation of their expression and 544 were down-regulated.

The *Stylophora pistillata* gene expression profile was analyzed with Partek Genomics Suite™ software (Fig. 3A) in a principal component analysis (PCA). The analysis showed that experiment replicates were profiled in the 3D PCA plot into two different groups—treated coral fragments (T11 and T23, which are fragments that were incubated in SW+100 mg/L at 11:00 and at 23:00) and second group—reference coral fragments (C11 and C23, which are fragments that were incubated in natural SW at 11:00 and at 23:00).

The microarray analysis revealed that 2,768 transcripts were differentially expressed (fold > 2) across all of the treatment groups. Among the differentially expressed gene targets, 1924 were found comparing treatment corals versus reference corals from night-time (T23 vs. C23). Comparing treatment corals with reference coral from daytime (T11 vs. C11) revealed 1,957 differentially expressed genes. In addition, comparing both lists of treatments corals with reference corals from night-time (T23 vs. C23) and daytime (T11 vs. C11) 1,113 common genes were identified (Fig. 3B). Within the 811 unique differentially expressed genes during night-time (which did not overlap with daytime) 267 genes were up-regulated and 544 were down-regulated. Within the 844 unique differentially expressed genes during daytime (which did not overlap with night-time) 437 genes were up-regulated and 407 were down-regulated.

In order to focus on genes that share similar biological processes and correlate with the calcification process, the analysis continued for unique up-regulated gene transcripts and total up-regulated gene transcripts that were differentially expressed during daytime (437 genes for unique and 1,333 for total), and during night-time (267 genes for unique and 1,171 for total). The total (found also to be up-regulated during the “opposite” time point) and unique genes were clustered into biological processes descriptions with GeneMania website analysis portal.

**Figure 4:**
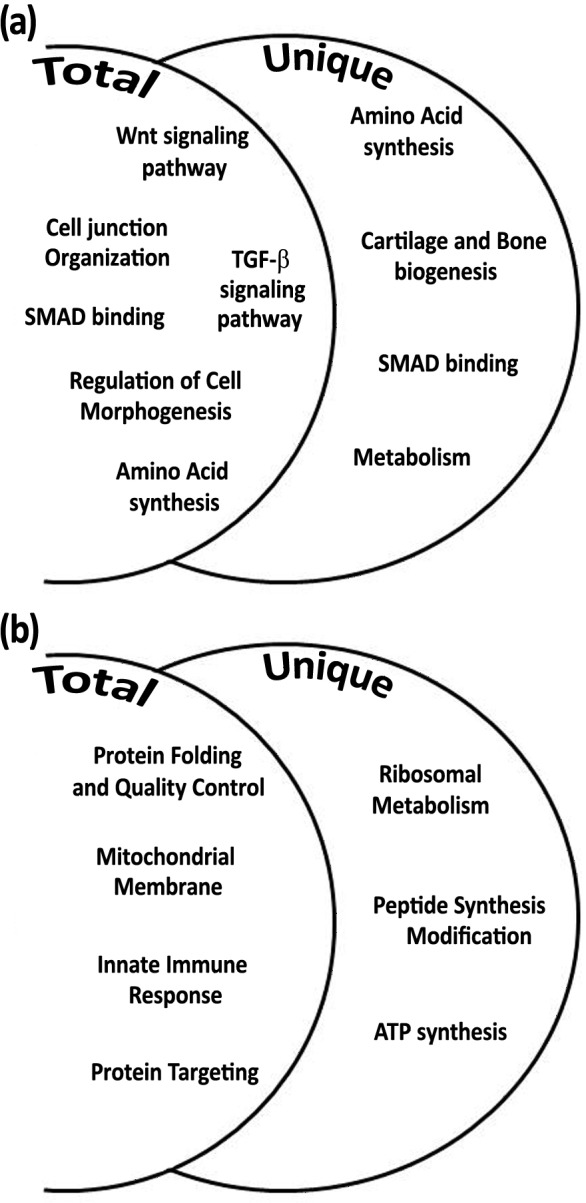
Biological processes according to differential expression of total and unique gene transcripts (genes that were up-regulated only during treatment) from treated vs. control during daytime (A), and from treated vs. control during night-time (B).

Among the total gene transcripts that were differentially expressed during daytime six biological processes were found: Wnt signaling pathway, cell junction organization, SMAD binding, regulation of cell morphogenesis, TGF-β signaling pathway, and amino acid synthesis (Fig. 4A). From the unique genes during daytime four biological processes were found: amino acid synthesis, metabolism, Smad signaling, and cartilage and bone biogenesis (Fig. 4A and Tables 2 and 3). All biological processes that were analyzed from total and unique genes were found with high significance of FDR *p*-value <0.05, except of “connective tissue development” cluster within the biological process cartilage and bone biogenesis, with FDR *p*-value of 0.068.

**Table 2:** List of biological processes and number of gene transcripts within each biological process, divided to daytime and night-time. All biological processes were found with FDR *p* < 0.05.

	*# gene transcripts*	*Biological process*
***Daytime (11:00)***		
Alpha-amino acid metabolic process	13	Amino Acid Synthesis
Alpha-amino acid catabolic process	9	
Cellular amino acid catabolic process	10	
Carboxylic acid catabolic process	11	Metabolism
Dicarboxylic acid metabolic process	6	
SMAD protein complex assembly	4	SMAD signaling
I-SMAD binding	4	
R-SMAD binding	4	
Cartilage development	10	Cartilage and Bone Biogenesis
Donnective tissue development^*^	7	
***Night-time (23:00)***		
Translational initiation	12	Ribosomal Metabolism
Large ribosomal subunit	9	
Ribosome	12	
Cytosolic large ribosomal subunit	8	
Translational elongation	10	
Cytosolic ribosome	9	
Translational termination	9	
Ribosomal subunit	10	
Structural constituent of ribosome	9	
Nuclear-transcribed mRNA catabolic process	10	
mRNA catabolic process	10	
Protein localization to endoplasmic reticulum	10	Peptide Synthesis Modification Processing
SRP-dependent cotranslational protein targeting to membrane	9	
Protein targeting to ER	9	
Cotranslational protein targeting to membrane	9	
Establishment of protein localization to endoplasmic reticulum	9	
Mitochondrial inner membrane	11	ATP synthesis & Oxidative Phosphorylation
Respiratory chain	6	
Respiratory electron transport chain	7	
Electron transport chain	7	
Mitochondrial ATP synthesis coupled electron transport	5	
ATP synthesis coupled electron transport	5	

**Notes.**

* *p* = 0.068.

**Table 3:** Fold change of genes identified for regulation of skeletal extension and growth, is presented as treated vs. control during daytime, night-time and without treatment. Their fold change *p*-values are indicated in brackets.

Clone ID	Annotation/Gene match name	Fold change C11 vs. C23 (*p*-value)	Fold change (T11 vs. C11) (*p*-value)	Fold change (T23 vs. C23) (*p*-value)	Functional classification
SMAD1	Mothers against decapentaplegic homolog 1	−1.5 (*ns*)	2.9 (1.6E−05)	1.7 (0.004)	SMAD protein complex assembly
SMAD2	Mothers against decapentaplegic homolog 2	−1.2 (*ns*)	2.7 (2.9E−07)	1.8 (5.1E−05)	
SMAD3	Mothers against decapentaplegic homolog 3	−1.1 (*ns*)	2.05 (1.5E−05)	1.4 (0.004)	
BMP1	Bone morphogenetic protein 1	−1.05 (*ns*)	2.3 (0.001)	1.8 (*ns*)	Cartilage & Bone Biogenesis
CTNNB1	Catenin (Cadherin-associated protein) beta−1	−1.06 (*ns*)	2.2 (0.001)	1.9 (0.006)	
COL2A1	Collagen alpha-1(II) chain	−2.04 (0.009)	2.1 (0.01)	−1.3 (*ns*)	
WNT5B	Protein Wnt-5b	−1.2 (*ns*)	2.3 (1.6E−05)	1.2 (*ns*)	
EIF2AK3	Eukaryotic translation initiation factor 2-alpha kinase 3	−1.08 (*ns*)	2.2 (2.9E−07)	1.7 (1.4E−05)	
ADAMTS2	A disintegrin and metalloproteinase with thrombospondin motifs 2	−1.05 (*ns*)	3.2 (2.9E−05)	1.4 (*ns*)	
ADAMTS7	A disintegrin and metalloproteinase with thrombospondin motifs 7	−1.3 (*ns*)	2.0 (1E−04)	1.05 (*ns*)	
LRP4	Low-density lipoprotein receptor-related protein 4	−1.1 (*ns*)	2.6 (9.5E−06)	1.8 (5E−04)	
FGFR1	Fibroblast growth factor receptor 1	−1.1 (*ns*)	2.1 (1.6E−06)	1.1 (*ns*)	
PAX7	Paired box 7 transcription factor	1.1 (*ns*)	2.3 (0.003)	1.4 (*ns*)	

**Notes.**

*ns* indicates for ‘not significant’.

Among the total gene transcripts that were differentially expressed during night-time four biological processes were found: protein folding, mitochondrial membrane, innate immune system, and protein targeting (Fig. 4B). From the unique genes during night-time three biological processes were found to be associated with ribosomal metabolism, peptide synthesis/modification/processing, and ATP synthesis and oxidative phosphorylation. (Fig. 4B and Tables 2 and 3). All night-time biological processes were found with high significance of FDR *p*-value <0.05.

(List of total genes for C11 vs. C23, T11 vs. C11, T23 vs. C23, and T11 vs. T23 can be found in File S2; Biological processes of these total gene transcripts analyses can be found in File S3).

**Figure 5:**
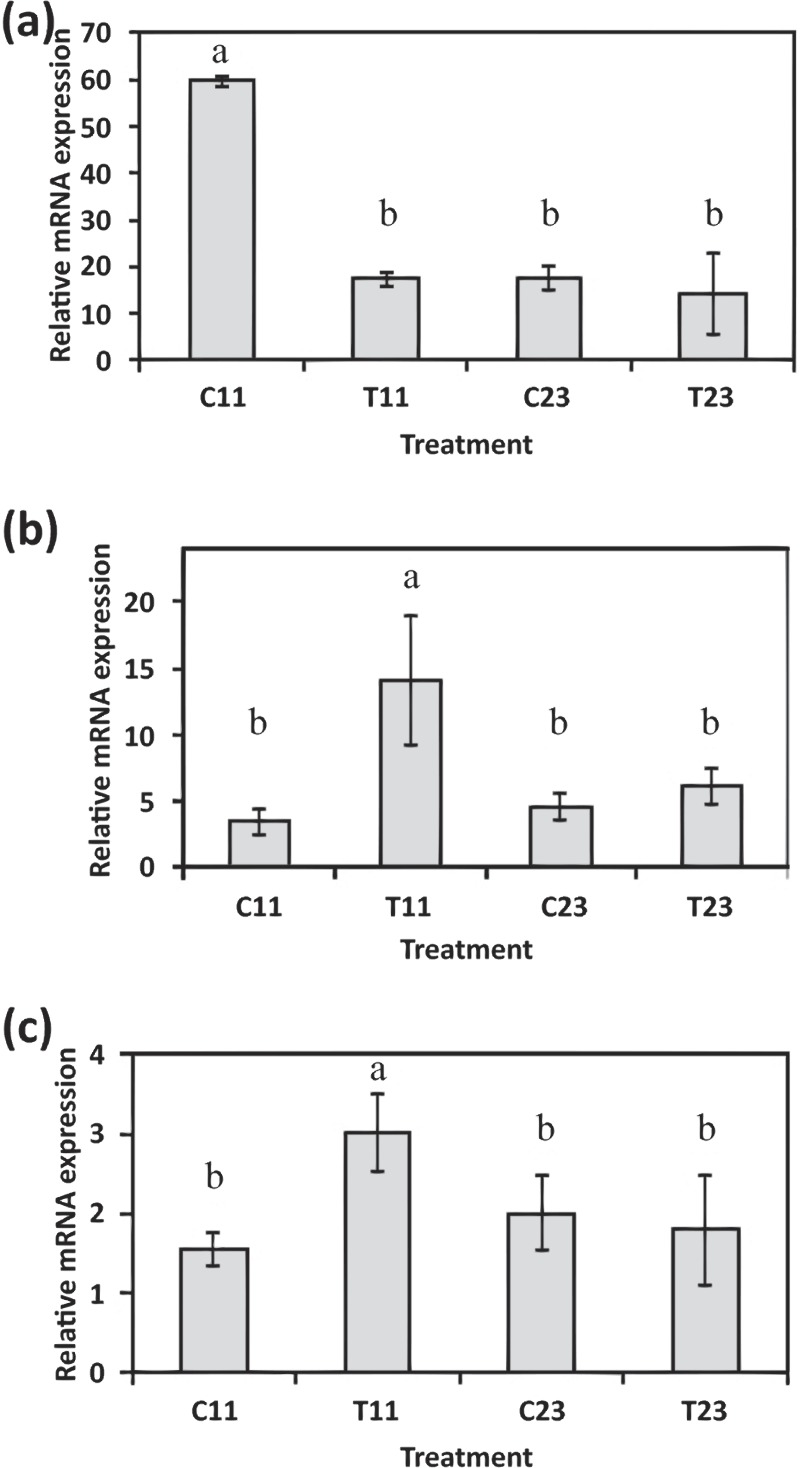
Relative mRNA expression results by semi-quantitative real-time RT-PCR for *S. pistillata*’s selected genes: *galaxin* (A), *galaxin-like* (B), and *smad1* (C). Relative gene expression was determined by the 2^−^ΔΔ^^ method and normalized by *β*-*actin*’s gene expression. Means followed by the same letter are not significantly different, one-way ANOVA followed by Tukey’s post-hoc test. Error bars represent SD. *n* = 3.

In order to expand the analysis of calcification associated genes, the gene expressions of *S. pistillata*’s orthologs of *galaxin* and *galaxin-like* were analyzed through semi-quantitative real-time PCR (Figs. 5A–5B and File S1). The *galaxin* gene has displayed a high relative expression during midday (relative expression of 59.76 ± 0.89 at 11:00, C11 in Fig. 5A) with statistically significant of *p* < 0.0005 (one-way ANOVA). While in treatment with addition of 100 mg/L calcium, relative expression of *galaxin* was dropped to expression level similar to expression during night-time (relative expression of 17.72 ± 2.61 at 23:00, C23 in Fig. 5A). The expression of gene *galaxin-like* was enhanced when treated with addition of 100 mg/L of calcium with relative expression of 14.14 ± 4.83 compared to relative expression of 3.48 ±  0.96 of the expression in the control corals at 11:00 (statistically significant *p* < 0.005, one-way ANOVA, *n* = 3). During night-time an addition of 100 mg/L calcium did not change *galaxin-like* gene expression (Fig. 5B).

Following the microarray analysis, the gene *smad1*, which was identified to be up-regulated during daytime and associated with the Smad signaling pathway was used to validate the microarray results (Fig. 5C). Semi-quantitative real-time PCR results of *smad1* displayed that its expression was elevated during the day to 3.00 ± 0.48 (T11 in Fig. 5C) when seawater had added calcium, compared to relative expression of 1.56 ± 0.2 in control corals (C11 in Fig. 5C; statistical significance *p* < 0.05, one-way ANOVA, *n* =3). However, there was no difference in the relative expression of the gene during the night-time (C23 and T23 in Fig. 5C).

## Discussion

Genetic pathways and regulatory networks are important for numerous cellular functions which enable the organism to survive in the environment through different physiological processes (Karlebach & Shamir, 2008).

Exposing a scleractinian coral to a seawater medium with high concentration of calcium ions caused an excessive expression of ortholog genes (Fig. 4A and 4B) which are assumed to be associated with processes of calcification, growth, and development (Tables 2 and 3). Moreover, using this approach we identified genes which are likely involved in cartilage and bone biogenesis in vertebrates.

Among the genes that were identified to be up-regulated are orthologs that encode for human collagen proteins: ADAMTS2, ADAMTS7 and COL2A1. These proteins are responsible for providing the infrastructure of a cartilaginous matrix for bone and cartilage growth in mammals. The cartilaginous matrix is composed of collagen and proteoglycans, and is generated by cartilage cells that are called chondrocytes. In addition, one of the genes identified—an ortholog of human COL2A1 is commonly used as a chondrocytic maker in the development of cartilage (Zaucke et al., 2001).

Another identified ortholog gene that emerged from the analysis is *bmp* (Bone Morphogenetic Protein) which is putatively associated with cartilage and bone promotion. The BMP protein acts as an activator for cell–surface receptors, and initiates cellular processes and signaling cascades. This protein also participates in another gene signaling pathway that was up-regulated in this study: the Smad signaling pathway. Smad genes are part of a transforming growth factor beta superfamily (TGF-β). The Smad genes are translated into multifunctional peptides, which play a role in the regulation of cell-type specification, proliferation, and differentiation of many cell types within a variety of species (Raftery & Sutherl , 1999; Sekelsky et al., 1995). Smad proteins are recruited after being phosphorylated by BMP receptors and assembled into a complex. The complex then migrates into the cell nucleus, and activates the transcription of specific target genes which are involved in physiological and pathological bone formation (Chen, Deng & Li, 2012; Katagiri & Tsukamoto, 2013; Miyazawa et al., 2002).

The ortholog genes that were found to be associated with cartilage development, along with *bmp* which acts as a key member gene, are known to be involved in the Wnt signaling pathway (Kim et al., 2013; Lin & Hankenson, 2011). This signaling pathway was found to be important in embryonic bone development (Wan et al., 2013), and in osteoblasts (bone cells) regulation (Glass et al., 2005; Harada & Rodan, 2003). The Wnt and TGF-β signaling pathways were identified in the gene expression analysis as being up-regulated during the day (Fig. 4). Lipid and nitrogen metabolism genes also exhibited up-regulation, which likely corresponds to the high metabolic requirements that support the coral growth and development. During the night, coral fragments treated with high calcium concentrations showed active genes that are involved in the mitochondrial synthesis of ATP and ribosomal metabolism. Ribosomal biogenesis and the translation of proteins included within their processing, folding and degradation have shown to be activated during the night and controlled by the circadian clock in past studies (Jouffe et al., 2013; Panda et al., 2002).

The high calcium concentration treatment during the day revealed genes associated with cell junction organization process exhibiting up-regulation (Fig. 4). Indication of re-organization of the cell junctions, which mediate adhesion and communication between tissues and cells may suggest a possible role in growth and development of the organism. Goldberg (2001) and Tambutté et al. (2007) have previously reported the occurrence of anchoring cells, also named desmocythe cells, which were suggested to secure the coral tissue to the extracellular matrix and/or to the skeleton.

Along with the cellular genes emphasized from the microarray analysis, two genes that are suspected to be involved in the scleractinian coral calcification process were tested for expression (Figs. 5A and 5B). These two genes in *S. pistillata*, *galaxin* and *galaxin–like*, were found to be similar to the *Acropora millepora*’s *galaxin* and *galaxin-like1* genes (Karako-Lampert et al., 2014), and have been characterized to be associated with the calcifying organic matrix (Fukuda et al., 2003). Both genes were affected by the calcium concentration treatment, during the day, but not at night. While *S. pistillata*’s *galaxin-like* gene was up-regulated during the daytime treatment, the *galaxin* gene was down-regulated, and was similar to night-time expression levels. These results are interesting because *galaxin* was first found due to its high levels in the organic matrix of scleractinian coral, and it was characterized as a soluble protein (Watanabe et al., 2003) and localized along the calcifying septa (Reyes-Bermudez et al., 2009). According to the findings of Reyes-Bermudez et al. (2009), the *galaxin-like* gene is considered to be a gene expressed only during planula stages (free swimming planula, pre-settled and post-settled planula in the case study of *A. millepora*), and not in the adult stages. Our results suggest that the role of galaxin genes in *S. pistillata* is possibly different from that in *A. millepora*.

**Figure 6:**
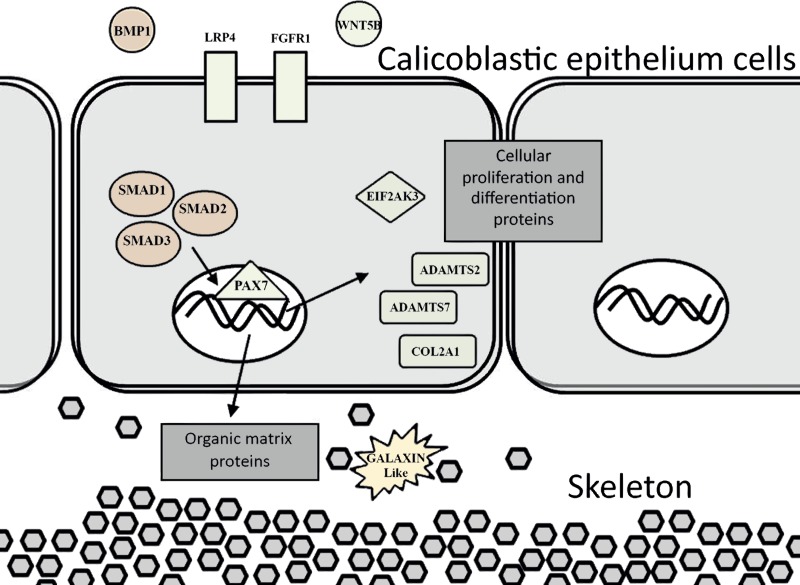
Proposed model for calicoblastic cells exhibiting cellular Wnt and TGF- β pathways during daytime calcification process. Members of Wnt are in green and TGF-β in orange.

The evidence of ortholog genes expression which likely associated with Smad proteins, Wnt, TGF-β signaling pathways, and cartilage formation in *S. pistillata* supports previous reports suggesting that gene regulatory pathways of development and biomineralization processes across the metazoan evolution are considerably conserved (Jackson et al., 2007; Marin et al., 2008). This study suggests that the Wnt signaling pathway cooperates with the TGF-β signaling pathway, similar to the mammalian cellular mechanism (Chen, Deng & Li, 2012). Both pathways appear to be responsible for the differentiation and proliferation of cell type similar to bone mass and osteoblasts (Fig. 6). Similarities between invertebrates and vertebrates regarding the elements associated with calcification process come in agreement with previous reports of Atlan et al. (1997) and Liao et al. (1997). Those studies showed that nacre implants from a bivalve mollusk were successfully incorporated and transplanted into a mammalian bone tissue due to similarity between the growth proteins present in nacre and those in bone. Similarity between the osteoblasts and the calicoblastic epithelium cells of scleractinian coral was also shown in the localization of the protein BMP in the tissue of *S. pistillata* (Zoccola et al., 2009), and for its role in the coral biomineralization (Mass et al., 2016; Zoccola et al., 2009).

Exposing *S. pistillata* to seawater with an elevated calcium concentration resulted in up-regulation of gene expression during the day relative to the night. In addition, the effect of the high calcium concentration was stronger in the calcification rates during day compared to the night (Fig. 2). Previous studies showed that the coral calcification process has a diel rhythmic cycle of increasing rates towards midday, and then decreasing towards dusk (Gutner-Hoch et al., 2016; Schneider et al., 2009). This could be an indication of a controlled expression of genes responsible for calcification during daytime, as evidenced by the up-regulation of genes associated with the cartilage and bone biogenesis process. Another possible explanation is the activity of the coral endo-symbionts during daytime which acts as important mean of energy to the coral’s cellular functions and physiology (Colombo-Pallotta, Rodríguez-Román & Iglesias-Prieto, 2010). Hence, it could be suggested that the small differences in the calcification rates observed between 100 mg/L and 200 mg/L calcium additions during the day could be a result of relatively mediocre photosynthetic activities of the endo-symbionts, but this should be further studied.

Uncovering regulatory genes involved in the scleractinian biomineralization process and their coded proteins is truly a challenge because genetic manipulation methods of mutagenesis are not yet accessible in scleractinian corals. However, the use of antagonists for the TGF-β/BMP and Wnt pathways associated with the coral calcification process has not been tested. Studying coral calcification with antagonists can provide further information and knowledge to better understand the role and function of genes that participate in the cellular control mechanism, and in the coral organic matrix.

##  Supplemental Information

10.7717/peerj.3590/supp-1 File S1 Calcification rates raw data

10.7717/peerj.3590/supp-2 File S2 DE genes microarray names and annotated names

10.7717/peerj.3590/supp-3 File S3 Biological process from the research analyses

## Additional Information and Declarations

### Field Study Permissions

The following information was supplied relating to field study approvals (i.e., approving body and any reference numbers): The Israeli Nature and National Parks Protection Authority approved the collection of corals in this study, permit No. 2009-35556.

### Data Availability

The following information was supplied regarding data availability: Our data has been submitted to the GEO repository (NCBI) under accession number GSE87159.
