# Supplementary material for: Identifying genes and regulatory pathways associated with the scleractinian coral calcification process
Source: PeerJ. 2017 Jul 20;5:e3590. doi: 10.7717/peerj.3590 (PMC5522607; doi:10.7717/peerj.3590)
Supplement: File S3 [file peerj-05-3590-s003.docx]

C11 vs C23:

| **Biological process** | **FDR** | **# gene transcripts** |
| --- | --- | --- |
| Mitochondrial ATP synthesis coupled electron transport | 0.016 | 6 |
| Endoplasmic reticulum lumen | 0.016 | 9 |
| Chaparone binding | 0.017 | 10 |
| Oxidaive phosphorylation | 0.036 | 6 |
| Cellular respiration | 0.059 | 8 |
| Translational elongation | 0.059 | 8 |

T11 vs C11:

| **Biological process** | **FDR** | **# gene transcripts** |
| --- | --- | --- |
| small conjugating protein ligase binding | 1.41 x 10^-7^ | 23 |
| SMAD binding | 1.3 x 10^-4^ | 11 |
| Extracellular matrix organization | 2.57 x 10^-4^ | 27 |
| Response to transforming growth factor beta | 7.14 x 10^-4^ | 19 |
| Cell junction organization | 1.73 x 10^-3^ | 19 |
| Cellular amino acid catabolic process | 2.26 x 10^-3^ | 11 |
| Wnt signaling pathway | 5.78 x 10^-3^ | 19 |
| Response to reactive oxygen species | 1.71 x 10^-2^ | 10 |

T23 vs C23:

| **Biological process** | **FDR** | **# gene transcripts** |
| --- | --- | --- |
| Response to unfolded protein | 1.32 x 10^-4^ | 18 |
| Protein targeting | 5.15 x 10^-4^ | 22 |
| Ubiquiting protein ligase binding | 1.49 x 10^-3^ | 16 |
| Response to endoplasmic reticulum stress | 2.91 x 10^-3^ | 14 |
| Mitochondrial membrane | 3.65 x 10^-3^ | 22 |
| Activation of innate immune response | 1.57 x 10^-2^ | 14 |

T11 vs T23:

| **Biological process** | **FDR** | **# gene transcripts** |
| --- | --- | --- |
| extracellular matrix organization | 9.63 x 10^-5^ | 16 |
| Water transport | 0.055 | 4 |
| Cellular modified amino acid metabolic process | 0.09 | 3 |
